# Multispectroscopic Characterization of Surface Interaction between Antibiotics and Micro(nano)-sized Plastics from Surgical Masks and Plastic Bottles

**DOI:** 10.1021/acsomega.2c07927

**Published:** 2023-03-28

**Authors:** Asli Baysal, Hasan Saygin

**Affiliations:** † Istanbul Technical University, Science and Letters Faculty, Chemistry Department, Maslak, Sariyer, Istanbul 34467, Turkey; ‡ Istanbul Aydin University, Application and Research Center for Advanced Studies, Sefakoy, Kucukcekmece, Istanbul 34295, Turkey

## Abstract

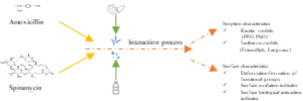

Recent studies have shown that plastic particles can sorb antibiotics, and these sorption properties have been examined in various studies; however, the possible mechanism responsible for the interactions requires a deeper investigation in terms of further interaction with living systems. Moreover, the usage of disposable surgical masks and plastic bottles has increased the plastic pollution risk for living systems like humans. Therefore, this study aimed to examine the sorption characteristics between antibiotics (amoxicillin and spiramycin) and plastic particles from surgical masks and plastic bottles through batch sorption experiments. In the study, their surface interactions were characterized using multispectroscopic approaches including FTIR, Raman spectrometry, and SEM-EDX, and various surface indicators (e.g., surface oxidation, deformation, and biological potential) were examined. The sorption results showed that adsorption kinetics and the isotherm of amoxicillin and spiramycin on micro(nano)plastics from surgical masks and plastic bottles closely fit the pseudo-second-order kinetic model and Langmiur isotherm. These results indicated that the evidence for the antibiotic interaction with particles was changes in the surface functional group intensities and up-shifting, and this correlated with the sorption of antibiotics on micro(nano)-sized plastics. The C/N ratio of the plastic particles before and after antibiotic treatment was used as an indicator for the surface biological interaction, and the results showed that C/N ratios of surgical mask particles increased with both types of antibiotic sorption. However, the C/N of the particles from plastic bottles showed antibiotic type-dependence. The surface deformation indicators (e.g., O/C, C=O, C=C, and O–H indices) showed that the O/C ratios of micro(nano)plastics from surgical masks were higher with the amoxicillin and spiramycin sorption, and the C=O indices were positively linked with the amoxicillin sorption stages, whereas the C=C and O–H had a negative correlation with the amoxicillin sorption stages. Moreover, amoxicillin sorption influenced the O/C ratio and indices of O–H and C=C of micro(nano)plastics from plastic bottles in a limited manner. The C=O groups of the micro(nano)plastics from plastic bottles were positively influenced by the spiramycin sorption stages, whereas it was negatively linked with amoxicillin sorption stages. Overall, the findings from surface indicators indicated that the micro(nano)plastics from surgical masks can be more influenced with antibiotic sorption compared to plastic bottles.

## 1 Introduction

The dramatically increasing production and utilization of plastic materials in different fields has led to its unavoidable presence in various environmental compartments and living systems, including human and plant life.^1^ Thus, the increasing abundance of plastic particles everywhere has raised serious concerns for both the environment and human health. These plastic particles are mainly classified by size as micro- (5 mm to 1 μm), submicron- (1 μm to 100 nm), and nano- (<100 nm) plastics.^2^ It is also known that the size of the particles affects their physicochemical properties and the decrease in the particle size may promote the surface area, reactivity, sorption, bioavailability, and biological impact on living systems.^3−7^ In the meantime, studies indicate that one of the main sources for human exposure to plastic particles may be via packaged water bottles including varied concentrations (11–6290 items, 0.4–11.4 μg/L) and sizes (<5 μm) of polyethylene terephthalate (PETE) plastic particles found within the packaging (e.g., bottle and/or cap).^8−12^ Moreover, the disposable face/surgical masks manufactured using nonwoven fabrics made from polypropylene (PP) plastics is another concern for the environment and human health due to their unavoidable use with the outbreak of coronavirus disease in 2019.^13^ Recent studies have reported that there were about 129 billion disposable face/surgical masks used every month in the world during the coronavirus disease in 2019.^14−16^ Several recent studies have also demonstrated that disposable face/surgical masks can release micro(nano)-sized plastics, additives, and heavy metals into the surrounding environment, and they could cause an adverse impact on both the environment and human health. The biological interaction or toxicity impact of micro(nano)plastics released from consumer/end-product plastic materials in living systems, specifically in humans, is yet to be well documented; however, there is evidence that indicated that micro(nano)-sized plastic particles could cause some illnesses like asthma, inflammation of the immune system, damage to some internal organs (e.g., the kidney), penetrate across membranes, and enter into the bloodstream.^17^

In addition, recent studies observed that plastic particles have been detected in the human placenta, the human lung, and human breast milk.^18−20^ These studies also observed that much bigger plastic particles than previously believed have been detected in the human body, specifically in lung-related studies, since it is known that <2.5 μm particles can enter the circulation according to general opinion. The previous studies showed that the length and width of plastic particles in lung tissue ranged between 12 and 2475 μm (mean: 223.10 ± 436.16 μm) and 4 and 88 μm (mean 22.21 ± 20.32 μm), respectively,^19^ and 13–125-μm-wide microfibers/plastics were found in the lung ground glass nodules.^20^ These studies also showed that the detected plastic particles in the human tissues mainly originated from PP and PETE types of plastics, which are similar polymers to those from surgical masks and plastic water bottles. In addition, previous studies also reported the occurrence of PETE and PP as the most common identified polymers in the atmospheric compartment.^21^

Presently, field and sorption studies showed that plastic particles sorbed or carried some chemical substances such as antibiotics into the surrounding environment.^22−27^ Although antibiotics can upgrade the life of living systems, overuse and misuse can cause serious environmental and health issues, including antibiotic resistance and contamination of the environment.^28^ In recent years, various laboratory-based studies approved the sorption of antibiotics onto microplastics. The antibiotic sorption properties onto plastic particles are considered to depend on the plastic particles’ surface chemistry, and various sorption pathways were described for the interaction of plastic particles with antibiotics, namely, the van der Waals force, hydrophobic interaction, electrostatic interaction, hydrogen bonding, partitioning, micropore-filling mechanism, π–π interaction, and charge-assisted hydrogen.^29−32^ In addition, it has also recently been confirmed that the antibiotic sorption capacity of micro(nano)plastics affects the further biological activity of the surfaces, e.g., biofilm attachment/production ability.^33^ Furthermore, the sorption of antibiotics onto micro(nano)plastics can cause another issue for the environment and human health, bacterial resistance. The sorption of antibiotics from the surrounding environment and selective colonization/enrichment of antibiotic-resistant bacteria on plastic particles exhibit favorable conditions for higher transmission and propagation of antibiotic-resistance.^34^ In addition, our recent research study demonstrated that the copresence of plastic materials and antibiotics lowered the inhibition efficiency of antibiotics on pathogens, and the copresence of antibiotics and micro(nano)plastics affected the formation of biofilms and extracellular polymeric substances.^35^ Therefore, it is vital to examine sorption characteristics and surface differences of plastic particles after the sorption of antibiotics due to understanding the further impact of antibiotic sorbed plastic particles on biological and chemical interactions like biofilm formation, antibiotic resistance, and cell interactions.

Although an increasing number of studies have investigated the interaction of plastic particles with other compounds (e.g., antibiotics) and their effect on the environment and human health, there are some critical problems in the laboratory-based studies in the literature. For example, most of the studies have examined sorption of antibiotics onto pure polymers; however, end-product plastics that contaminated both the environment and humans include various additives and residual nonpolymerized monomers compared to pure polymers. These additives (e. g., plasticizers; metals as catalysts, antiblocking agents, compatibilizers, diluents, flame retardants, heat or UV stabilizers, antimicrobials, antioxidants, and pigments) can significantly influence their properties (sorption, degradation, etc.).^36−38^ Some recent studies have highlighted the fact that the toxicity of plastic particles in the environment was different from the toxicity of pure polymers.^39,40^ Moreover, the aging/weathering of plastics and the generation rate of plastic particles change with their chemical composition and the chemical composition of the surrounding environment.^40^ Thus, all of these studies have been underlining the importance and applications of consumer or end-product plastics, such as plastic bottles or surgical masks that are more appropriate material than pure polymers for their realistic interactions in the environmental and human-related process. However, there is limited information using consumer or end-product plastics with their sorption of antibiotics.^28^

Moreover, the physiochemical changes of the consumer micro(nano)-sized surgical masks and bottles due to their interactions with other environmentally or biologically related contaminants and compounds (e.g., antibiotics) could be important for understanding the effects of consumer plastic materials after sorption on the environment and on human health, since the sorption of antibiotics on micro(nano)plastics is very complex and the exposed micro(nano)plastic surface would continuously change. The changes of the micro(nano)plastic surface due to sorption processes can influence their sorption capability, result in crystallinity, and change morphology (e.g., formation of cracks).^41,42^ Therefore, clarifying the surface differences during the sorption process would contribute to understanding the interaction between micro(nano)plastics and antibiotics that controls the sorption, tendency, and dominant behavior and then biological interactions.

Therefore, in the current study, the batch sorption kinetics of antibiotics on micro(nano)plastics and the surface characteristics of micro(nano)plastics before and after treatment of antibiotics were examined. For this purpose, a more realistic proxy was chosen for the experiments which used consumer/end-products such as plastic water bottles and surgical masks to obtain micro(nano)sized-plastic particles and applied two commercial antibiotics (amoxicillin and spiramycin) for medical use. To understand the underlying surface interaction mechanism of micro(nano)plastics with antibiotics and the further impact of antibiotic sorbed micro(nano)plastics, the surface functional groups; elemental distribution; and deformation, oxidation, and biological activity indicators of micro(nano)plastics were examined before and after the sorption of antibiotics using various exposure stages.

## 2 Materials and Methods

### 2.1 Materials

The amoxicillin (AMOX) and spiramycin (SPM) antibiotics were commercially obtained; further information is given in Supporting Information Table S1 and Supporting Information Figure S1. Commercially available plastic drinking water bottles (DW) and surgical masks (SM) were obtained from markets in Istanbul, Turkey to prepare micro(nano)plastics. Ultrapure water was obtained using a Milli-Q water purification system (Merck, Darmstadt, Germany; conductivity 0.055 μS/cm at 25 °C, pH 6.9).

### 2.2 Preparation of Micro(nano)plastics

The preparation of micro(nano)plastics was based on our previous studies.^35,38^ Bottles and masks were first rendered with a stainless-steel render on a clean bench. The particles were filtered with a <5 μm stainless-steel sieve and washed several times using ultrapure water and then dried. Throughout the study, <5 μm mesh sized plastic particles were used. The prepared plastic particles were characterized using surface functional groups, elemental composition, surface area, size distribution, and charge by Fourier transform infrared–attenuated total reflection spectroscopy (FTIR–ATR, Bruker InvenioS ATR), Raman spectrometry (Thermo, DXR Raman), scanning electron microscopy–energy dispersive X-ray spectroscopy (SEM/EDX, QUANTA FEG 250, FEI, Thermo Fisher Scientific, Oregon, USA), Brunauer–Emmett–Teller surface area by the multipoint measurement (BET, Micromeritics Gemini VII 2390t), and dynamic light scattering (DLS, Zetasizer Nano ZS, Malvern Instruments, UK).

### 2.3 Sorption of Antibiotics on Micro(nano)plastics

Batch experiments of micro(nano)plastics for the sorption of antibiotics (AMOX and SPM) in the aqueous system were performed in triplicate, and the results were reported as the mean values. The sorption experiments were carried out at room temperature and ay pH 7.0. The control group or blank group used the same method in the same sorption system but without micro(nano)plastics or antibiotics (AMOX and SPM), respectively.^43,44^

In the kinetic experiment, 10 mg of micro(nano)plastics was added to a glass tube filled with 10 mL of 0.6 mg/L AMOX and SPM. The solution was shaken at 150 rpm, and the micro(nano)plastics were collected after 1, 2, 4, 8, 24, and 48 h. The change in the concentration of AMOX and SPM was determined using a UV–vis spectrophotometer, UV–VIS 96-well plates (Thermo Scientific MULTISKAN GO, Finland).

The sorption kinetics (e.g., pseudo-first-order (PFO) and pseudo-second-order (PSO)) are given by the following formula:
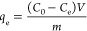
1
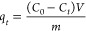
2where *C*_0_ (mg/L) represents the original concentration, *C*_e_ (mg/L) represents the concentration at equilibrium, *C*_t_ (mg/L) represents the concentration at a given time, *V* (L) and *m* (g) respectively represent the volume of the antibiotic solution and the mass of the micro(nano)plastics, and *q*_e_ (mg/g) and *q*_*t*_ (mg/g) represent the sorption capacity at equilibrium and at a given time, respectively.
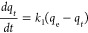
3
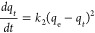
4

Equation 3 is the PFO model, *q*_e_ (mg/g) is the sorption capacity at equilibrium, and *q*_*t*_ (mg/g) is the sorption capacity at a given time. Equation 4 is the PSO kinetics model. *k*_1_ is the rate constant of the PFO kinetics model, and *k*_2_ is the rate constant of the PSO kinetic model.

For the sorption isotherms, we selected different concentrations of AMOX and SPM (0.1–28.0 mg/L). The same mass of micro(nano)plastics was transferred to each tube, and the equilibrium time was determined from the kinetics experiments. The equilibrium was reached at 8–24 and 24 h for AMOX and SPM, respectively. Therefore, the isotherms were conducted at 24 h. The sorption isotherms (e.g., Langmuir and Freundlich) are given by the following formula:
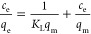
5
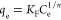
6In the Langmuir adsorption isotherm eq 5, *c*_e_ is the equilibrium concentration of the antibiotic; *q*_e_ is the adsorption capacity of the antibiotic onto micro(nano)plastics; *q*_m_ is the maximum adsorption capacity; and *K*_L_ is the Langmuir adsorption constant. The value of *K*_L_ is higher, and the adsorption ability is stronger.

Equation 6 is the Freundlich sorption isotherm. *K*_F_ is the Freundlich adsorption constant. *n* is the Freundlich adsorption index, and the common range of *n* is from 0 to 10. When *n* > 1, the adsorption is preferential.

### 2.4 Characterization the Micro(nano)plastics: before and after Antibiotic Interaction

For the characterization of micro(nano)plastics before and after antibiotic antibiotic interaction, the particles were washed with ultrapure water and dried under a vacuum for 1 week before analysis.^45−48^ The measurements were repeated at least three times from the same portion in the batch and at least three times from different portions in the same batch.

Particle size distribution and charge of the nontreated plastic particles was analyzed in ultrapure water using dynamic light scattering (DLS, Zetasizer Nano ZS, Malvern Instruments, UK).^45−48^

The surface area of the nontreated plastic particles was tested using the Brunauer–Emmett–Teller surface area by the multipoint measurement (BET, Micromeritics Gemini VII 2390t). The BET specific surface area measurement was performed by krypton adsorption at 77 K. The micro(nano)plastics were degassed for 24 h at 90 °C prior to analysis to ensure removal of impurities for pores of the plastic samples.^45^

The antibiotic treated and nontreated micro(nano)plastics were further characterized by surface functional groups and elemental composition. The sample preparation was similar to that in the batch adsorption studies. The antibiotic treated micro(nano)plastics were used at various exposure stages so that the higher sorptions were obtained and compared to the nontreated micro(nano)plastics. After antibiotic treatment, each batch was removed from the antibiotic solution, washed with ultrapure water, and dried under a vacuum for 1 week before analysis. The dry-treated micro(nano)plastic particles were portioned as at least three parts from the same batch; the measurements were repeated at least three times using a separate portion of each batch, as well as the same portion, and directly tested using FTIR and Raman spectrometry. Surface functional group characteristics were examined through FTIR-ATR (Bruker InvenioS ATR) and Raman spectrometry (Thermo, DXR Raman).^4−6^ The FTIR-ATR and Raman spectrometry were also utilized in the 4000–400 cm^–1^ range. The FTIR analysis was conducted with 64 repetitive scans and a resolution of 4 cm^–1^.^49^ To further analyze the functional groups, three likely areas of deformation-related difference in the FTIR spectrum were identified, and functional group indices were calculated according to studies by Brandon et al. and Stark and Matuana.^50,51^ The carbonyl (C=O), hydroxyl (OH), and vinyl (C=C) indexes were calculated as the absorbance ratios for the C=O, OH, and C=C detection wavelengths at approximately 1720, 3360–3860, and 910 cm^–1^, respectively, and the reference peak wavelength (alkane CH stretching vibrations of the methylene groups).^50−52^

Raman spectroscopy was conducted with a 532 nm excitation laser and 100× objective lens with a ∼1 μm laser spot size, 600 g/mm, and 3 s acquisition time.^45,53^

The elemental distribution of micro(nano)plastics was analyzed using a SEM/EDX spectrometer.^4−6,45−48,53^ The ratios of elemental oxygen to carbon (O/C) and carbon to nitrogen (C/N) were calculated from the EDX spectra.

### 2.5 Statistical Analysis

The differences between the control and samples, as well as the differences among samples, were analyzed via ANOVA with post hoc Tukey (*p* < 0.05). SPPS 17.0 software was applied for the significance and Spearman correlation (two-tailed) tests.

## 3 Results and Discussion

### 3.1 Characterization of Micro(nano)-sized Plastics before Sorption Process

**Figure 1:**
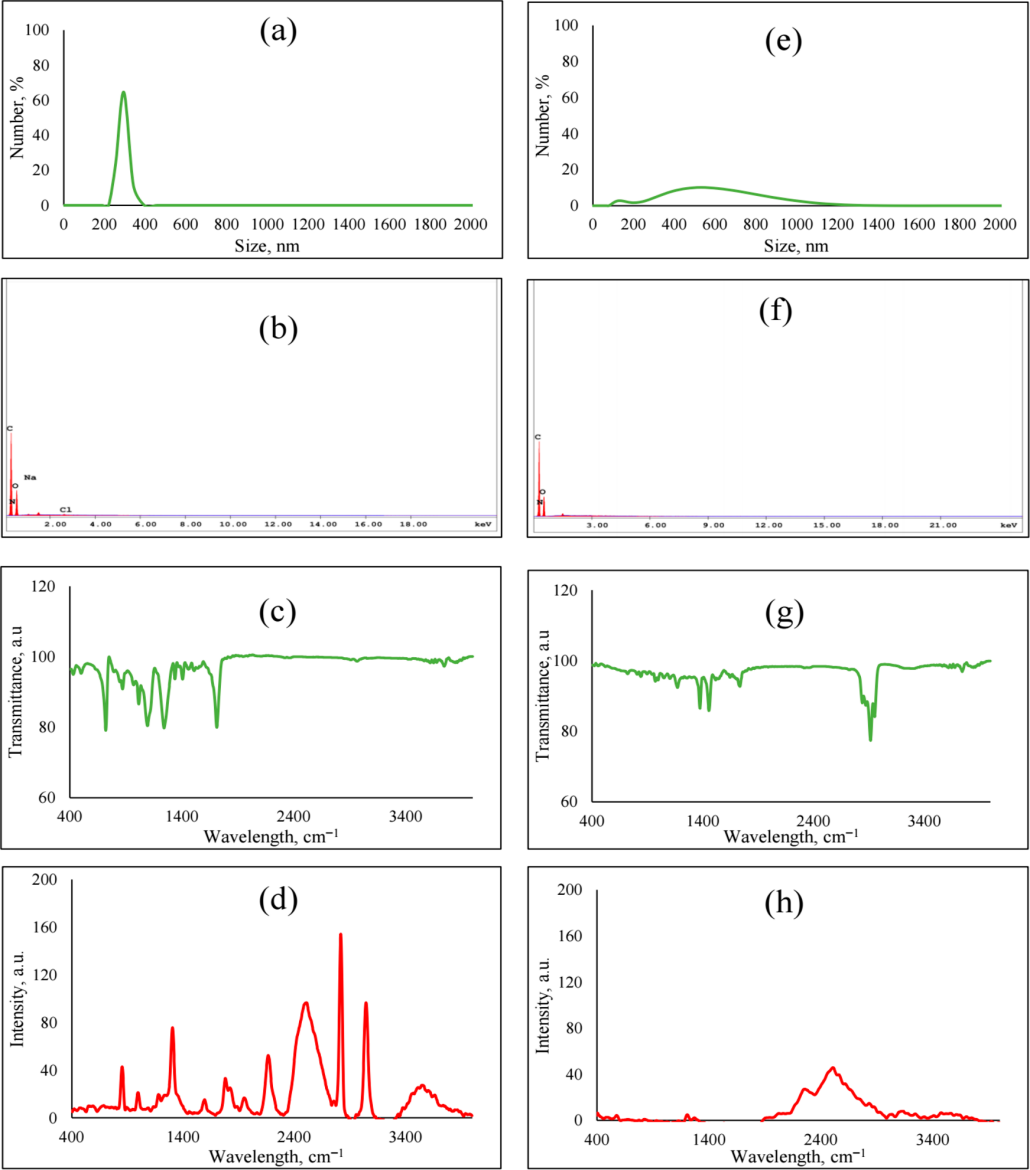
Characterization of plastic water bottle and surgical mask particles. (a) Size distribution, (b) elemental composition, (c) FTIR, and (d) Raman spectrum of plastic particles obtained from plastic water bottles. (e) Size distribution, (f) elemental composition, (g) FTIR, and (h) Raman spectrum of plastic particles obtained from surgical masks.^35^

The surface characteristics of plastic particles obtained from plastic bottles and surgical masks were characterized by DLS, EDX, FTIR, and Raman spectrometry as shown in Figure 1 and Table 1. The size distribution results indicated that the particle sizes were changed between 220 and 396 nm and 296 and 1292 nm for plastic bottles and surgical masks, respectively (Figure 1a and e). Moreover, as indicated in Table 1, the zeta potentials and surface area of the plastic particles were measured as 12.9 ± 0.2 mV and −1.7 ± 0.6 mV and 0.3257 m^2^/g and 0.9095 m^2^/g for plastic bottles and surgical masks, respectively.^35^

**Table 1:** Zeta Potentials and Surface Area of the Plastic Particles Obtained from Plastic Water Bottles and Surgical Masks

parameter	zeta potentials, mV	BET surface area, m^2^/g
plastic bottles	12.9 ± 0.2 mV	0.3257
surgical masks	–1.7 ± 0.6 mV	0.9095

Additionally, elemental composition of the plastic particles using SEM–EDX was shown in Figure 1b and f, and they indicated that the PETE particles obtained from plastic bottles mainly include carbon, oxygen, nitrogen, sodium, and chloride. On the other hand, the surgical mask particles (PP) contain only carbon, oxygen, and nitrogen.

FTIR spectra of original plastic particles obtained from plastic bottles and surgical masks are illustrated in Figure 1c and g. The absorption peak near 1710 cm^–1^ is caused by the carbonyl (C=O) group, whereas 1240 and 1090 cm^–1^ and 720 cm^–1^ indicated that the C–O stretch and aromatic C–H out-of-plane bend corresponded to polyethylene terephthalate (PETE; Figure 1).^35,54^ The FTIR spectra of particles of surgical masks are shown in Figure 1b. It can be clearly observed that the peaks at approximately 2950 cm^–1^, 2915 cm^–1^, 2838 cm^–1^, 1455 cm^–1^, 1377 cm^–1^, and 1166 cm^–1^ are attributed to the C–H stretch, C–H stretch, C–H stretch, CH_2_ bend, CH_3_ bend, CH bend, CH_3_ rock, C–C stretch, and indicated polypropylene (PP).^54^ Furthermore, the Raman spectra of original plastic particles are shown in Figure 1d and h. The Raman spectra were identified as PETE based on the peaks of C=O, C–O stretch, and C–H at 2800–2900 cm^–1^ and between 850 cm^–1^ and 1110 cm^–1^ and were also verified by FTIR. Similarly, PP was identified by several peaks at 2800–3000 cm^–1^ in the Raman spectrum, but the peaks of the CH_2_ stretching vibration between 2800 and 3000 cm^–1^ in the spectra were very broad.

### 3.2 Sorption of Antibiotics on Micro(nano)plastics

**Figure 2:**
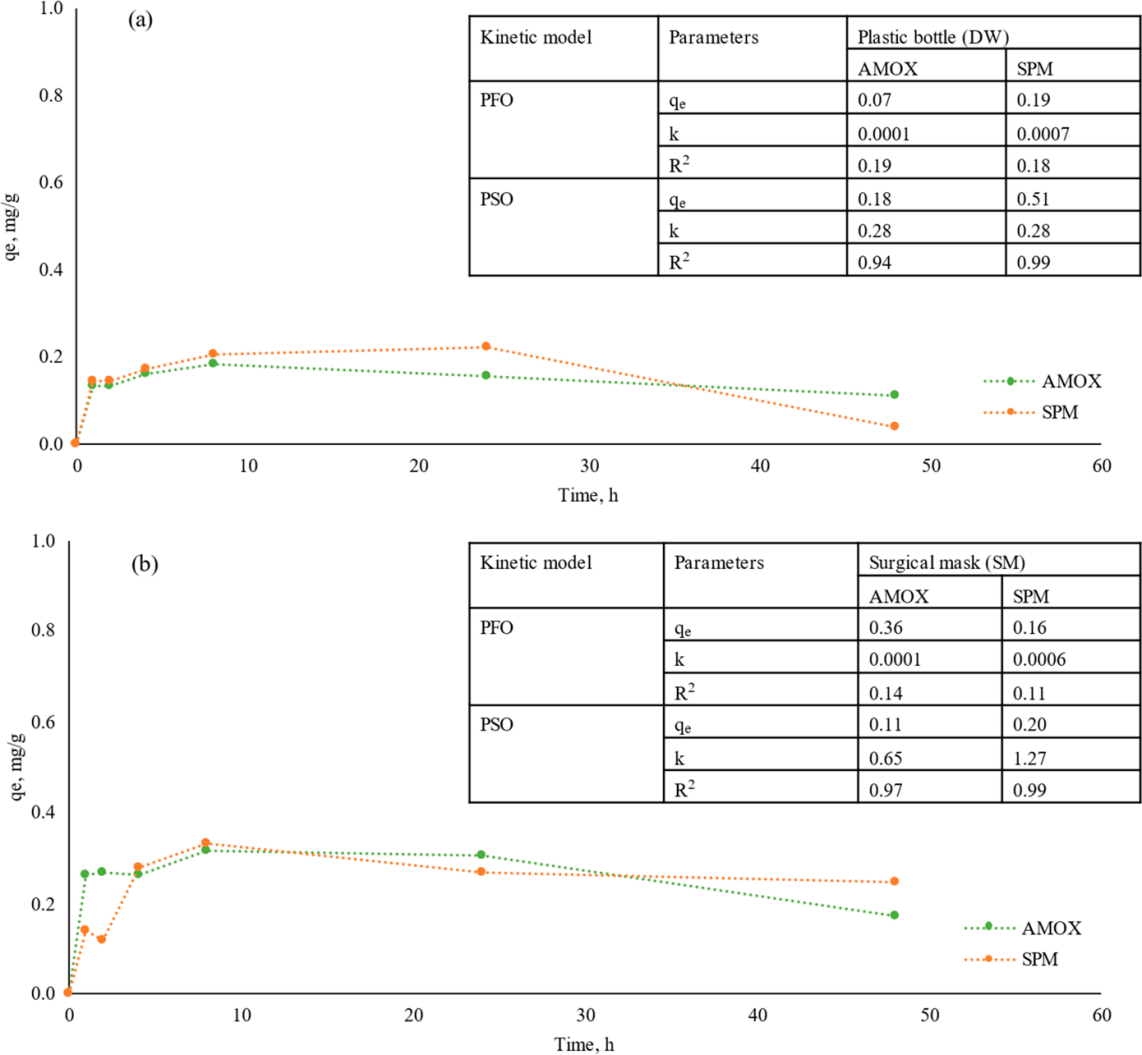
Sorption kinetics. (a) Sorption capacity of AMOX and SPM on micro(nano)plastics from plastic water bottles. (b) Sorption capacity of AMOX and SPM on micro(nano)plastics from surgical masks. Embeded tables are model fitting kinetics parameters of antibiotics (AMOX and SPM) sorption on micro(nano)plastics from plastic water bottles and surgical masks. DW, plastic water bottles; SM, surgical masks; AMOX, amoxicillin; SPM, spiramycin.

The sorption kinetics were analyzed using the PFO and PSO models, and the results are presented in Figure 2. Compared with the PFO model, the PSO model can better describe the process of the two antibiotics adsorbed by micro(nano)plastics from plastic bottles and surgical masks. This result is also in a good agreement with the other antibiotic sorption studies on various polymer types.^55^ Sorption kinetics are also mainly used to describe the sorption rate of the adsorbate by the sorbent.^25,32^ As shown in Figure 2, the sorption equilibrium was achieved after at least 24 h. During the first 4 h, the sorption capacity increased, and higher sorption was obtained between 4 and 24 h. Similar sorption equilibrium durations have also been observed in various studies in the literature.^42,56^ The kinetic coefficient *k* for the AMOX and SPM on micro(nano)plastics from surgical masks was greater compared to the micro(nano)plastics from plastic bottles, which suggested that the sorption rate decreased dramatically in micro(nano)plastics from plastic bottles. The lower *k* values of the micro(nano)plastics from plastics bottles indicated that the sorption rate is proportional to the number of unoccupied sites, and the sorption sites of micro(nano)plastics from plastic bottles are rapidly occupied by antibiotics, resulting in a decline in *k*.^55^ Furthermore, the sorption kinetic results showed that greater *q*_e_ values were obtained with SPM sorption on both micro(nano)plastics.

**Figure 3:**
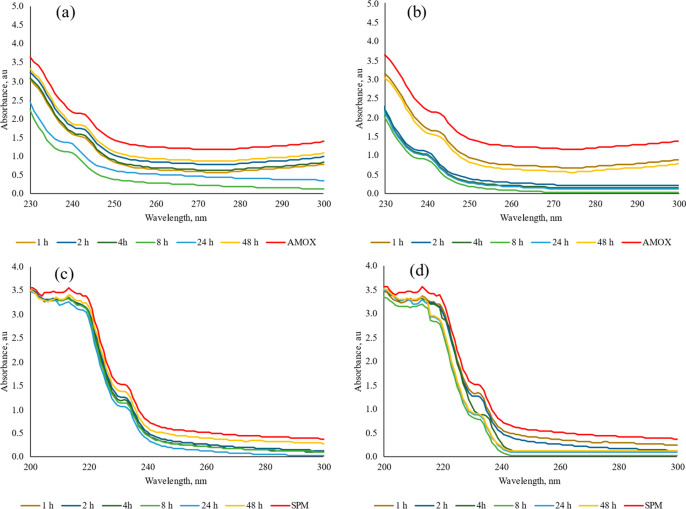
Interaction of antibiotics (AMOX and SPM) with micro(nano)plastics from drinking water bottles and surgical masks for 1, 2, 4, 8, 24, and 48 h. (a) AMOX solution before and after the interaction with micro(nano)plastics from plastic water bottles. (b) AMOX solution before and after the interaction with micro(nano)plastics from surgical masks. (c) SPM solution before and after the interaction with micro(nano)plastics from plastic water bottles. (d) SPM solution before and after the interaction with micro(nano)plastics from surgical masks.

The sorption of antibiotics onto micro(nano)plastics has been investigated by means of UV–vis spectroscopy. The spectra of antibiotic solutions were taken before and after the micro(nano)plastics treatment (Figure 3). As seen in Figure 3, it can be found that with the treatment time, the intensity of the absorption peaks decreases. The decrease of the intensity of the absorption peaks is due to the decrease of concentration of AMOX and SPM with the sorption on micro(nano)plastics.^57^ The absorption spectrum for the AMOX solution before micro(nano)plastics exhibits an electronic absorption band around 230 and 250 nm.^58^ Upon contact with the micro(nano)plastics, the absorption band sligthly shift which is parallel with sorption capacity (Figure 3a,b). With the increase of sorption onto micro(nano)plastics, the absorbance declined and the peak position was weakly blue-shifted. This indicated that the interaction between micro(nano)plastics and AMOX may produce a new ground state complex, thus changing the UV–vis spectra of the system.^59^ However, the shift was not observed with the sorption of SPM onto micro(nano)plastics (Figure 3c,d).

**Figure 4:**
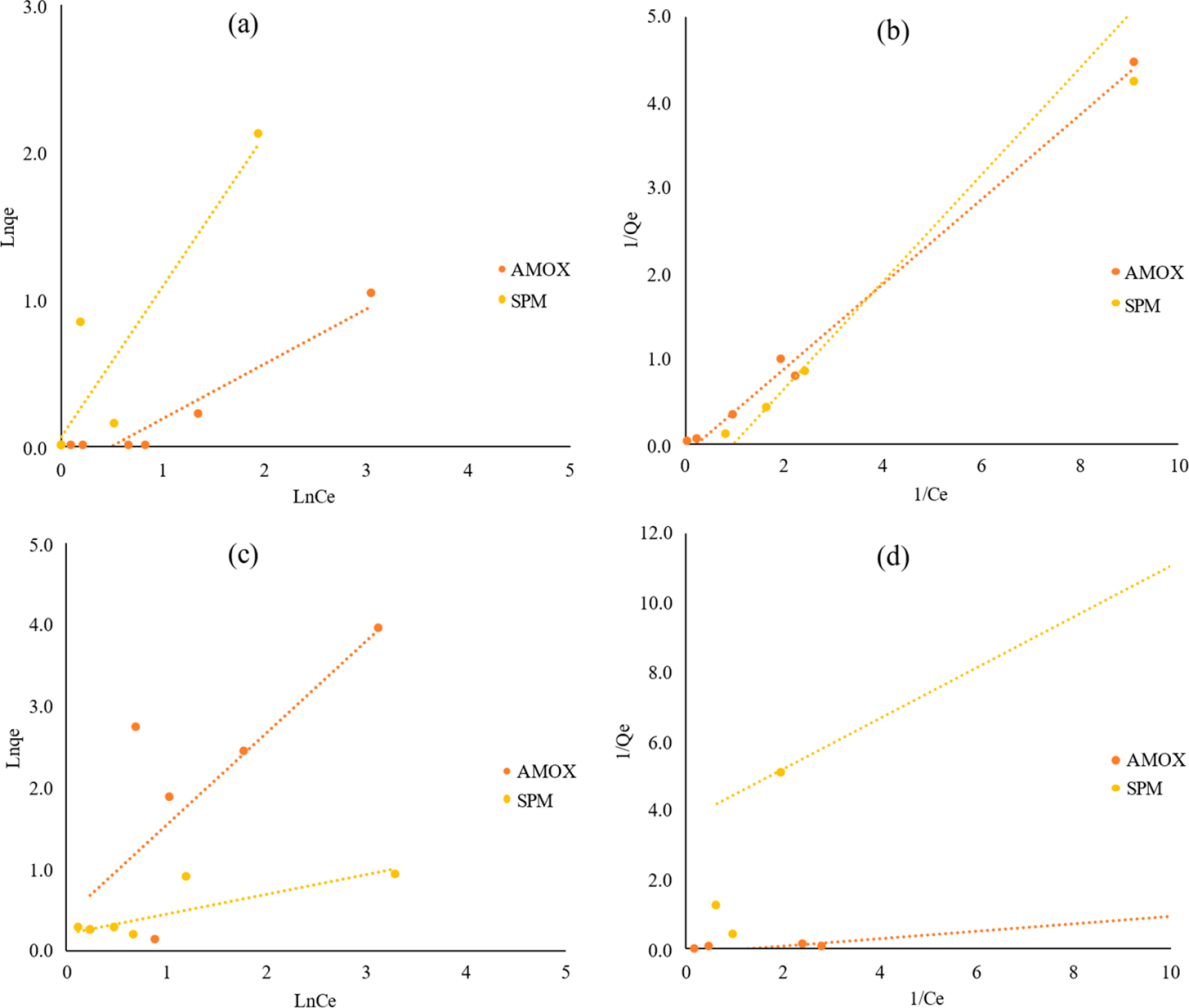
Sorption isotherms. (a) Freundlich and (b) Langmiur isotherms of AMOX and SPM on micro(nano)plastics from plastic water bottles. (c) Freundlich and (d) Langmiur isotherms of AMOX and SPM on micro(nano)plastics from surgical masks. DW, plastic water bottles; SM, surgical masks; AMOX, amoxicillin; SPM, spiramycin.

The sorption isotherms were described using common models such as Freundlich and Langmuir to characterize the interactions between antibiotics and micro(nano)plastics from plastic bottles and surgical masks.^60−64^Table 2 and Figure 4 show the Freundlich and Langmuir isotherm models. Based on *R*^2^, the Langmuir model is more suited to the sorption of AMOX and SPM on the PETE micro(nano)plastics from plastic water bottles. The suitability in the Langmuir model revealed that the sorption occurred through a homogeneous surface and containing active sites.^60−64^ The calculated *R*_L_ values of the Langmuir models were all between 0 and 1, indicating that the sorption is favorable.^60^ On the other hand, the Freundlich isotherm model also has higher *R*^2^ values that show that the sorption of antibiotics is suitable on micro(nano)plastics from plastic water bottles. The Freundlich model also shows that sorption occurs on heterogeneous surfaces with multiple layers and multiple sites.^60−64^ The significant parameter of the Freundlich “*n*” indicating the intensity of sorption showed that the values were *n* > 1, which remarked on the sorption processes occuring via physical sorption.^60−64^ The sorption experiment results for micro(nano)plastics from surgical masks indicated that Freundlich is more suitable for AMOX sorption, whereas the Langmuir model is appropriate for SPM sorption on micro(nano)plastics from surgical masks. The results also indicated that *q*_m_ is higher in the sorption of SPM on both types of micro(nano)plastics compared to AMOX sorption.

**Table 2:** Regression Parameters of Sorption Isotherms of Antibiotics (AMOX and SPM) onto Micro(nano)plastics Obtained from Plastic Bottle and Surgical Mask Particles by Freundlich and Langmuir Models (DW, plastic bottles; SM, surgical masks; AMOX, amoxicillin; SPM, spiramycin)

		plastic bottle (DW)		surgical mask (SM)	
isotherm	parameter	AMOX	SPM	AMOX	SPM
Freundlich	*K*_F_	1.48	1.16	2.63	1.65
	*n*	5.9	1.0	2.4	4.2
	*R*^2^	0.92	0.84	0.61	0.67
Langmuir	*K*_L_	9.4 × 10^–01^	1.8 × 10^–01^	5.1 × 10^0^	1.3 × 10^00^
	*q*_m_	1.7	10.99	0.3	7.01
	*R*_L_	0.002	0.01	0.0004	0.002
	*R*^2^	0.96	0.99	0.27	0.90

### 3.3 Impact of the Antibiotic Sorption on Surface Characteristics of Micro(nano)plastics

**Figure 5:**
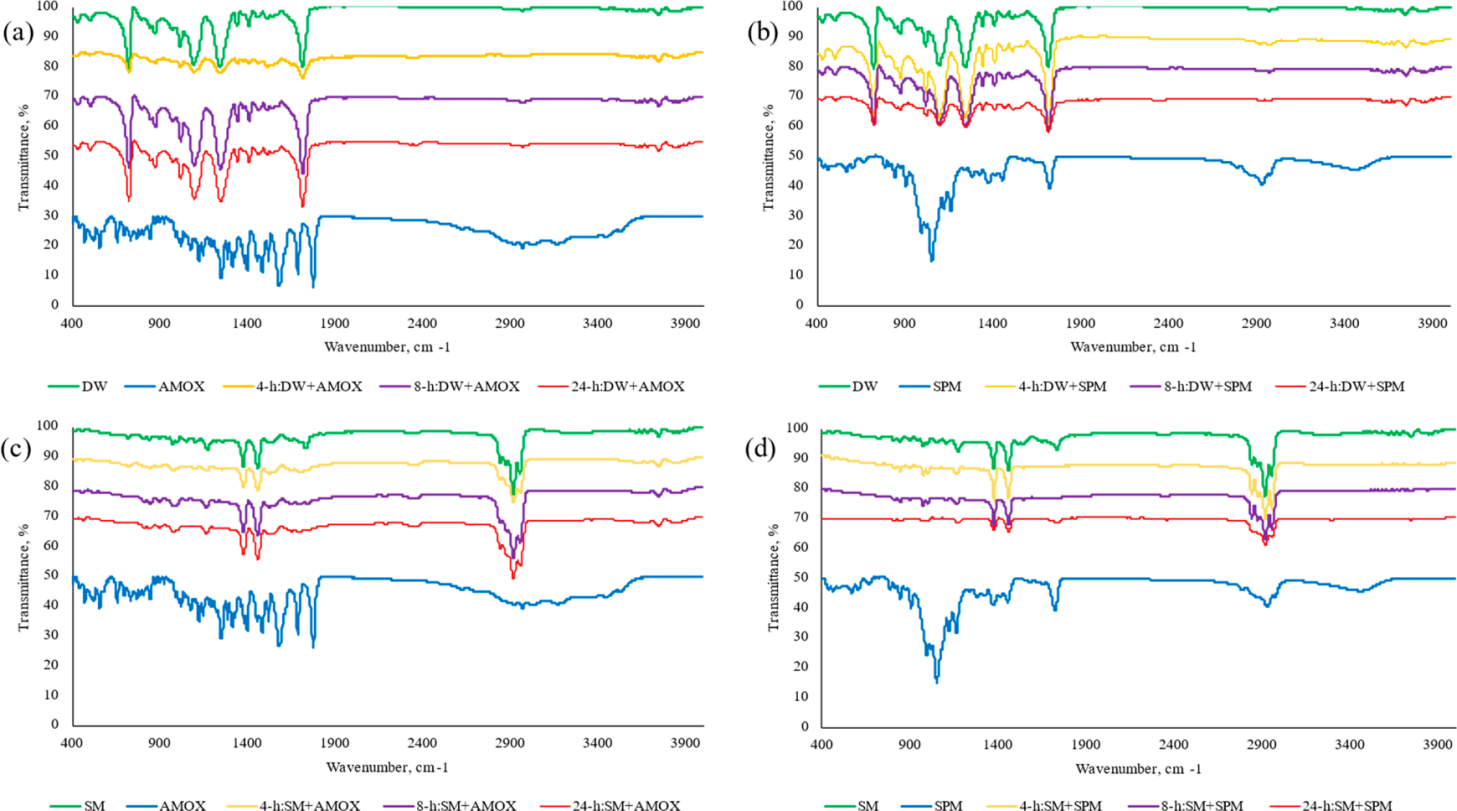
FTIR spectra of antibiotics (AMOX and SPM) and micro(nano)plastics before and after antibiotic sorption. (a) AMOX sorption on micro(nano)plastics from plastic water bottles, (b) SPM sorption on micro(nano)plastics from plastic water bottles, (c) AMOX sorption on micro(nano)plastics from surgical masks, and (d) SPM sorption on micro(nano)plastics from surgical masks. DW, plastic water bottles; SM, surgical masks; AMOX, amoxicillin; SPM, spiramycin.

To characterize the surface interactions of micro(nano)plastics from plastic bottles and surgical masks after the antibiotic sorption, the micro(nano)plastics were used at various exposure stages so that the higher sorptions were obtained. The FTIR spectra of original and antibiotic-treated micro(nano)plastics are illustrated in Figure 5. For micro(nano)plastics from plastic bottles, the absorption peak near 1710 cm^–1^ (C=O), 1240 and 1090 cm^–1^ (C–O stretch), and 720 cm^–1^ (aromatic C–H out-of-plane bend) was similar in the antibiotic-treated micro(nano)plastics; however, the peak intensities changed (Figure 5a,b). This result was consistent with those of the antibiotic-treated plastic particles, which indicated the antibiotic sorption onto microplastics.^43,55^ The results also showed that the peak intensities correlated with the sorption of antibiotics on micro(nano)plastics. Previous studies have indicated that changes in the intensities, specifically the oxygen-containing groups, might affect the hydrophilicity of treated particles.^65^ Moreover, the main peaks corresponding to C=O, C–O, and C–H were shifted with the exposure of antibiotics, which indicated the interaction between micro(nano)plastics from plastic bottles and antibiotics. Similarly, in micro(nano)plastics from surgical masks, the peaks approximately at 2950 cm^–1^, 2915 cm^–1^, 2838 cm^–1^, 1455 cm^–1^, 1377 cm^–1^, and 1166 cm^–1^ attributed to PP particles were indicated after antibiotic sorption on PP particles (Figure 5c,d); however, the intensities of the peaks were influenced corresponding to the sorption of antibiotics. After the sorption of AMOX and SPM, the bands on the PP micro(nano)plastics were up-shifted to long wavenumbers. This upshift suggested the interaction between plastic particles and antibiotics that was observed for the hydrophobic interactions between antibiotics and PP particles.^66^

**Figure 6:**
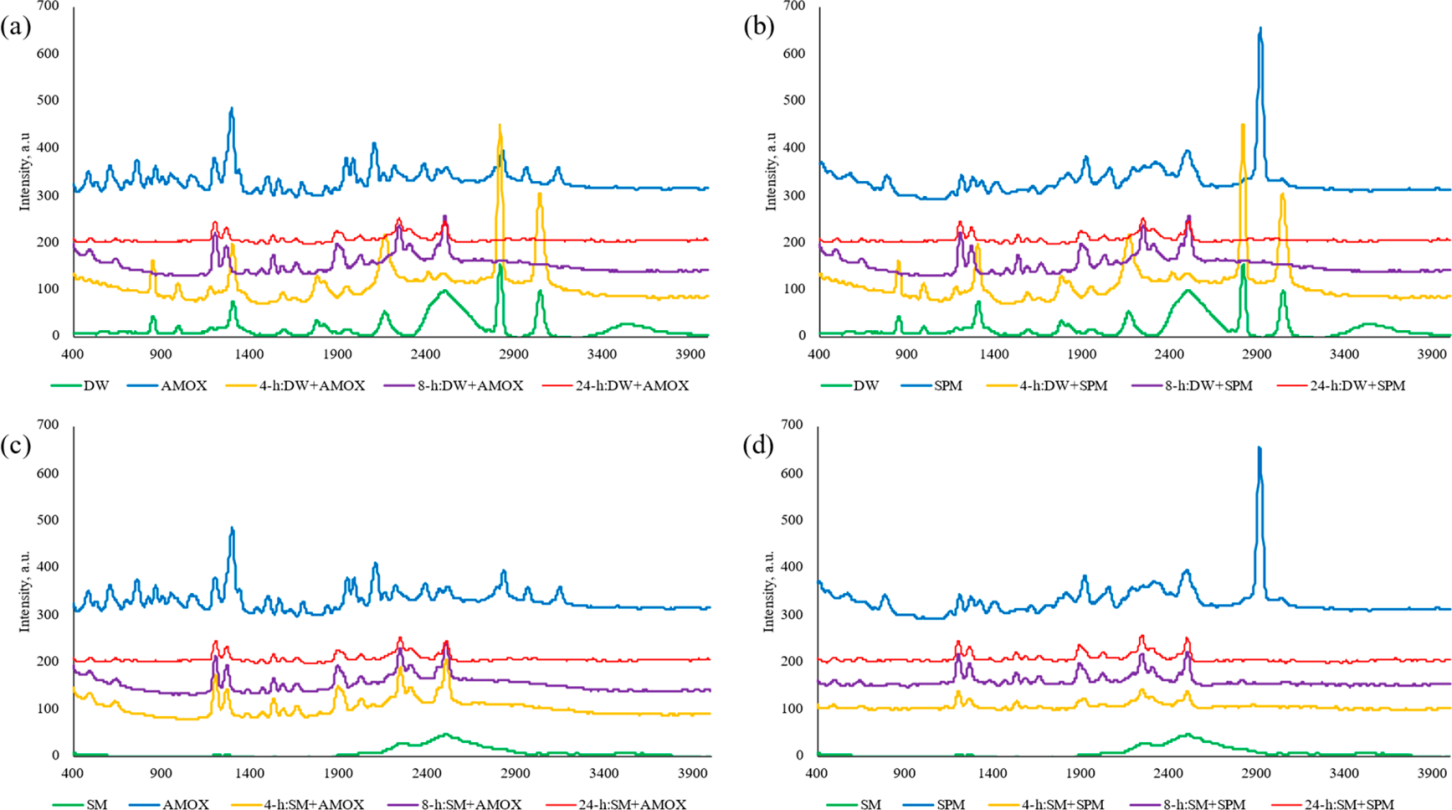
Raman spectra of antibiotics (AMOX and SPM) and micro(nano)plastics before and after antibiotic sorption. (a) AMOX sorption on micro(nano)plastics from plastic water bottles, (b) SPM sorption on micro(nano)plastics from plastic water bottles, (c) AMOX sorption on micro(nano)plastics from surgical masks, and (d) SPM sorption on micro(nano)plastics from surgical masks. DW, plastic water bottles; SM, surgical masks; AMOX, amoxicillin; SPM, spiramycin.

The Raman spectra of the antibiotic-treated micro(nano)plastics also show the interaction between AMOX and SPM and micro(nano)plastics (Figure 6). Since Raman spectroscopy is more sensitive to unsaturated groups than FTIR spectroscopy.^67^ For micro(nano)plastics from plastic bottles, the peaks of C=O at 2800–2900 cm^–1^ were removed with the antibiotic treatment, and new peaks appeared with the impact of antibiotics, as shown in Figure 6a,b. In addition, the C–O stretch and C–H peaks between 850 cm^–1^ and 1110 cm^–1^ were up-shifted under the impact of the antibiotics. For the micro(nano)plastics from surgical masks, broad peaks at 2600–3000 cm^–1^ in the Raman spectrum corresponding to CH_2_ stretching vibrations were separated and became more distinct with the impact of the antibiotics, which indicated the interaction between PP micro(nano)plastics and antibiotics (Figure 6c,d). Furthermore, the Raman intensities were also changed with the treatment of antibiotics, and this finding was correlated to the sorption of antibiotics on the micro(nano)plastics.

**Figure 7:**
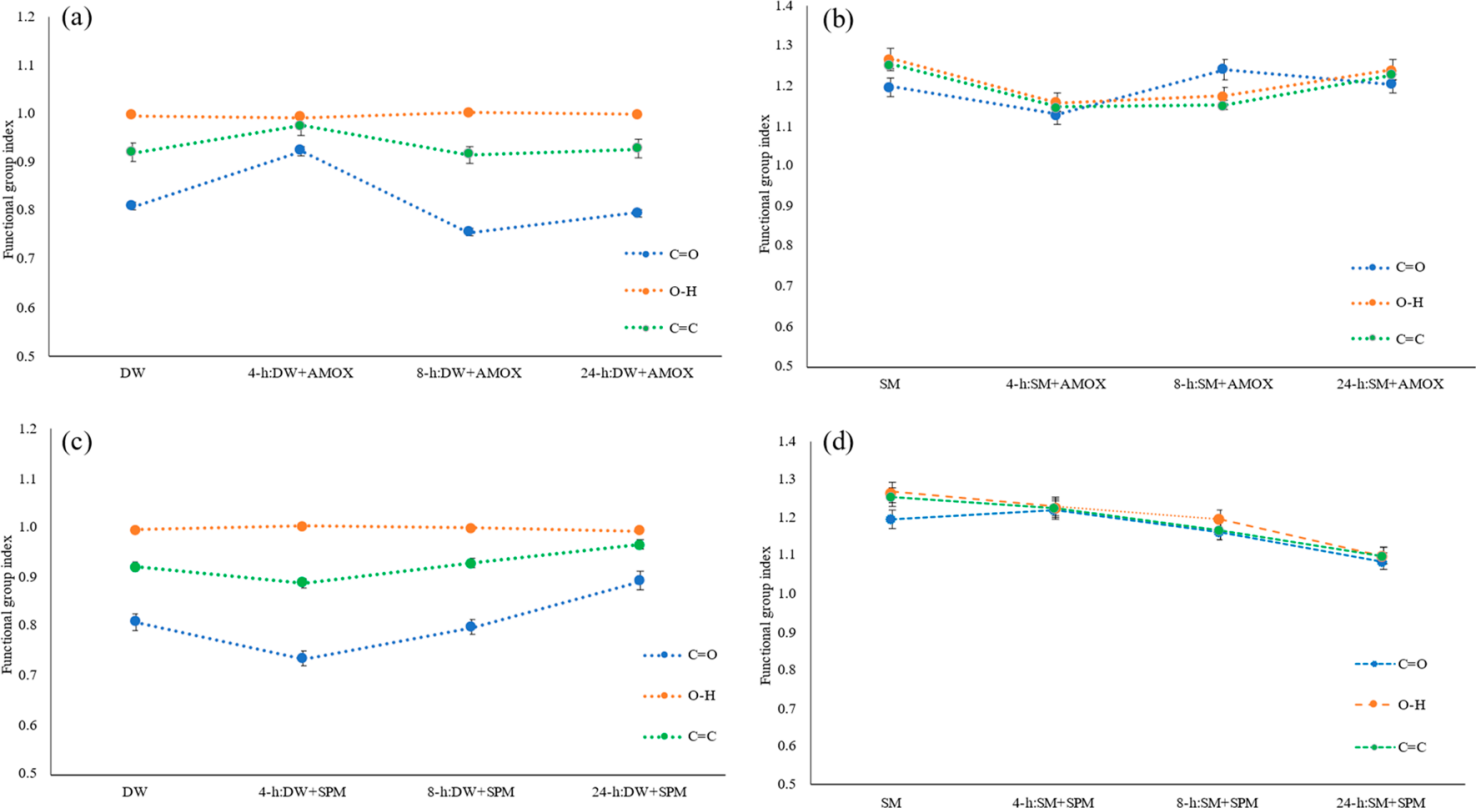
C=O, O–H, and C=C indices before and after the sorption of antibiotics on micro(nano)plastics. (a) AMOX sorption on micro(nano)plastics from plastic water bottles, (b) AMOX sorption onto micro(nano)plastics from surgical masks, (c) SPM sorption on micro(nano)plastics from plastic water bottles, and (d) SPM sorption on micro(nano)plastics from surgical masks. DW, plastic water bottles; SM, surgical masks; AMOX, amoxicillin; SPM, spiramycin.

To further measure the FTIR changes in the original and antibiotic-treated micro(nano)plastics, the C=O, O–H, and C=C indices were used and illustrated in Figure 7. These functional groups also indicated the level of weathering/aging or deformation levels on the plastics.^38^ According to Figure 4, the results indicated that the O–H groups on the plastic bottle micro(nano)plastics before and after the antibiotic treatment were not changed. However, other deformation related groups (C=O and C=C) were affected by the antibiotic sorption. The C=O and C=C of micro(nano)plastics from plastic bottles were negatively correlated with the AMOX sorption, whereas the SPM sorption positively linked the C=O and C=C indices of micro(nano)plastics from plastic bottles. The highest of the C=O and C=C indices with the AMOX sorption obtained at the early exposure duration (4 h) were compared to the untreated particles. This result could indicate that the functional groups on the untreated (original) micro(nano)plastics might be removed or deformed with the early exposure duration (4 h) of AMOX; however, with the increasing exposure duration, the surface could be modified by the C=O and C=C groups with the sorption of AMOX. Conversely, this result is different in the sorption of SPM on the micro(nano)plastics from plastic bottles, and C=O and C=C groups were positively linked with SPM sorption. The different behavior with the antibiotic type might be explained in that SPM modification can be started in more early exposure stages due to the density or charge differences. For the C=O, O–H, and C=C indices of micro(nano)plastics from surgical masks, different responses were obtained compared to the micro(nano)plastics from plastic bottles. For instance, the C=O indices were not significantly changed with AMOX sorption; however, the C=O indices decreased at early exposure compared to untreated micro(nano)plastics, and then they increased with increasing sorption duration compared to untreated micro(nano)plastics and the early SPM-sorbed one. This result indicated surface modification and then the possibility of deformation with the AMOX during the interaction with micro(nano)plastics from plastic bottles. On the other hand, SPM sorption had a slight and positive link with O–H, C=O, and C=C groups of the micro(nano)plastics from surgical masks. These deformation-related functional groups had similar behavior and declined with the sorption duration. These results also suggested that the surface modification of micro(nano)plastics from surgical masks with the antibiotic interaction was varied compared to micro(nano)plastics from plastic bottles.

**Figure 8:**
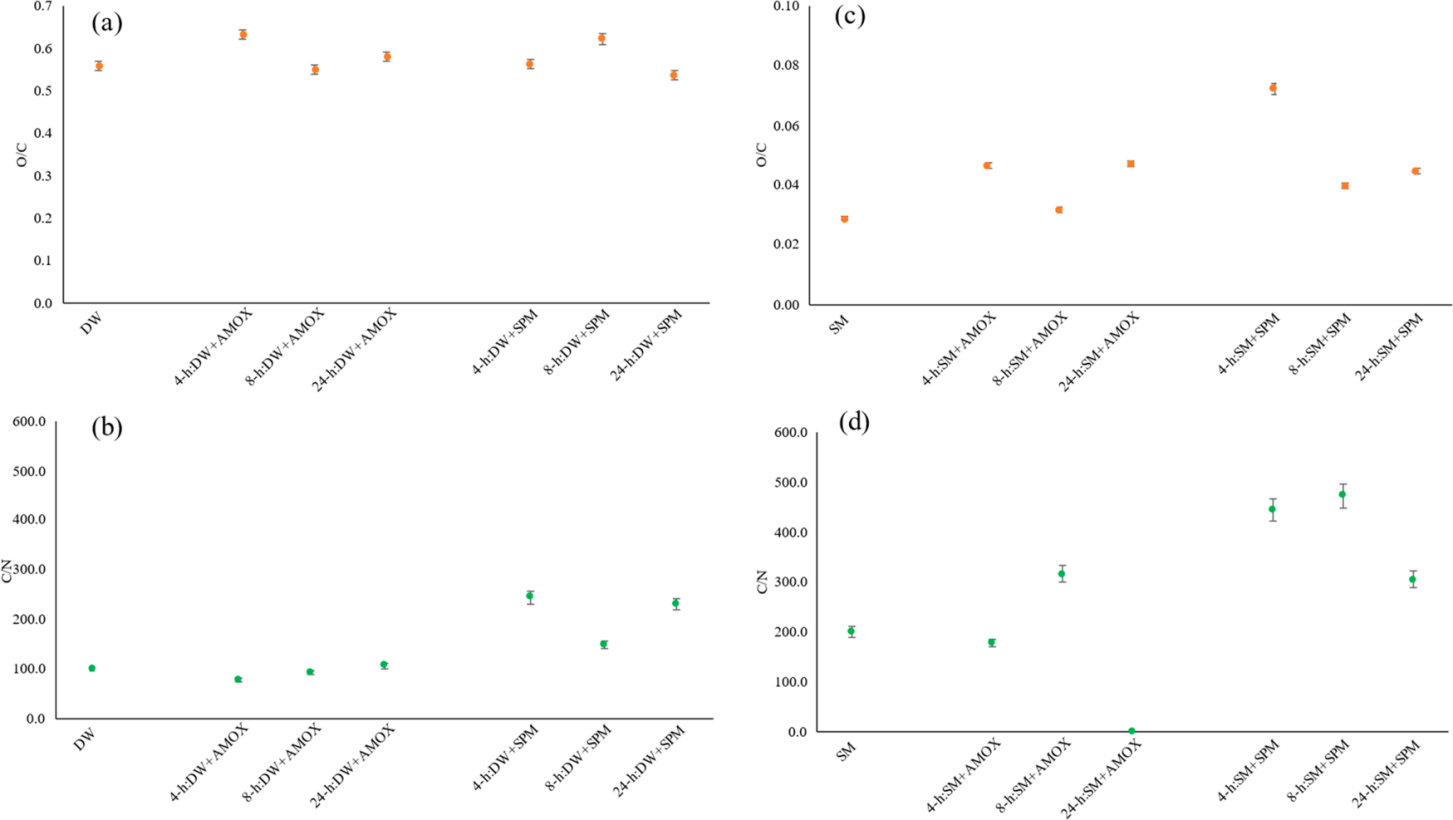
O/C and C/N ratio before and after the sorption of AMOX and SPM on micro(nano)plastics from plastic water bottles and surgical masks. (a) O/C ratio of micro(nano)plastics from plastic water bottles before and after AMOX and SPM sorption. (b) C/N ratio of micro(nano)plastics from plastic water bottles before and after AMOX and SPM sorption. (c) O/C ratio of micro(nano)plastics from surgical masks before and after AMOX and SPM sorption. (d) C/N ratio of micro(nano)plastics from surgical masks before and after AMOX and SPM sorption. DW, plastic water bottles; SM, surgical masks; AMOX, amoxicillin; SPM, spiramycin.

Additionally, for further evaluation between micro(nano)plastics and antibiotics, the O/C and C/N ratios of the original and AMOX- and SPM-sorbed micro(nano)plastics was calculated by EDX spectra. The O/C ratio of the polymer shows the surface oxidation state. According to Figure 8a and c, the O/C ratios of micro(nano)plastics from plastic bottles was negatively correlated with the AMOX sorption; however, there was positive correlation with the SPM sorption. On the other hand, limited changing was observed compared to the untreated micro(nano)plastics from plastic bottles. The highest O/C ratios were obtained at early (4 h) AMOX sorption and medium (8 h) SPM sorption of micro(nano)plastics from plastic bottles. Moreover, the O/C ratios of antibiotic-sorbed micro(nano)plastics from surgical masks were negatively linked with the AMOX and SPM sorption, and higher O/C ratios were obtained after the antibiotic sorption, indicating the polymer oxidation.^38^ These results suggested that the micro(nano)plastics from surgical masks are more susceptible for the polymer deformation/modification, even if negatively influenced by the sorption. Contrarily, the micro(nano)plastics from plastic bottles are more resistant to the surface deformation by antibiotic exposure. Similar results were reported in previous studies, where the O/C of polyethylene microplastics increased with an increase in treatment duration time with AMOX.^43,55^

The C/N ratios might be an important indicator, since the C/N ratio can be linked with microbial growth, production of extracellular polymers, residue decomposition, sorption of cations, and remineralization in the interaction between soil and natural substances (e.g., biochar).^68−70^ However, there has been no study regarding the C/N values in microplastics after the interaction with any substances or various aging processes (e.g., UV, heat). Therefore, we also calculated the C/N ratio of the micro(nano)plastics before and after antibiotic sorption. As illustrated in Figure 8b and d, the C/N ratios of untreated (original) and antibiotic-treated micro(nano)plastics showed that there was slight positive correlation between C/N ratio and the sorption of AMOX on the micro(nano)plastics from plastic bottles during the sorption process. Moreover, C/N ratios of AMOX-sorbed micro(nano)plastics from plastic bottles were not significantly changed compared to untreated particles, but C/N ratios of SPM-sorbed micro(nano)plastics from plastic bottles were greater compared to untreated particles; this result also negatively linked with the O/C ratio and sorption stages of SPM-sorbed micro(nano)plastics from plastic bottles. For the micro(nano)plastics from surgical masks, the C/N ratios were positively correlated with the sorption of both antibiotics. In addition, the C/N ratios of SPM-sorbed micro(nano)plastics from surgical masks were greater compared to the original. However, the C/N ratios of micro(nano)plastics from surgical masks were affected to a limited extent at the early sorption stage of AMOX compared to the later stages, and dramatic changes were observed with the increasing sortion stages, which might be explained by the surface functional groups and O/C ratios.

## 4 Conclusion

**Figure 9:**
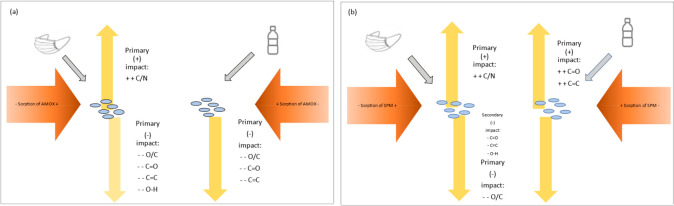
Summary of possible interaction between micro(nano)plastics from plastic water bottles and surgical masks and antibiotics. (a) AMOX and (b) SPM.

In this study, sorption experiments and surface properties of micro(nano)plastics from plastic bottle and surgical mask particles were examined after the sorption of two types of antibiotics (AMOX and SPM). The findings showed that sorption is observed according to the kinetic and isotherm characteristics. The results also indicated that the surface interaction between micro(nano)plastics and antibiotics can be variable according to the polymer and antibiotic types. Furthermore, micro(nano)plastics from surgical masks and plastic bottles exhibited different behaviors under antibiotic treatment considering the deformation indicators (Figure 9). The micro(nano)plastics from plastic bottles were more selective in the surface deformation/formation process according to the type of antibiotic and exposure duration compared to surgical masks. Since the C=O groups of micro(nano)plastic from plastic bottles significantly affected from antibiotic sorption, however, other deformation and oxidation indicators in the micro(nano)plastics from surgical masks were influenced from the antibiotic sorption. This study also investigated the C/N ratio, which might be correlated with the biological activities, and the micro(nano)plastics from surgical masks showed consistent results in this parameter with the sorption of antibiotics. Moreover, the findings could be important since the interaction between antibiotics and micro(nano)plastics has a primary impact on the human health–pathogen relationship.

## Supporting Information Available

(Table S1) Information for selected antibiotics. (Figure S1) Characterization of the antibiotics: (a) FTIR spectrum of amoxicillin, (b) Raman spectrum of amoxicillin, (c) EDX spectrum of amoxicillin, (d) FTIR spectrum of spiramycin, (e) Raman spectrum of spiramycin, (f) EDX spectrum of spiramycin (PDF)
