# Supplementary material for: Multispectroscopic Characterization of Surface Interaction between Antibiotics and Micro(nano)-sized Plastics from Surgical Masks and Plastic Bottles
Source: ACS Omega. 2023 Mar 28;8(14):12739–51. doi: 10.1021/acsomega.2c07927 (PMC10099137; doi:10.1021/acsomega.2c07927)

Supporting Information

Multispectroscopic characterization of surface interaction between antibiotics and micro(nano)-sized plastics from surgical masks and plastic bottles

Asli Baysal [a]* and Hasan Saygin[b]

[a] Istanbul Technical University, Science and Letters Faculty, Chemistry Dept. Maslak Sariyer, Istanbul, 34467, Turkey.

[b] Istanbul Aydin University, Application and Research Center for Advanced Studies, Sefakoy Kucukcekmece, Istanbul, 34295, Turkey

Corresponding author e-mail: asli.baysal@itu.edu.tr

Table captions

Table S1. Information for selected antibiotics

Figure captions

Figure S1. Characterization the antibiotics: (a) FTIR spectrum of amoxicillin, (b) Raman spectrum of amoxicillin, (c) EDX spectrum of amoxicillin; (d) FTIR spectrum of spiramycin, (e) Raman spectrum of spiramycin, (f) EDX spectrum of spiramycin

Table S1. Information for selected antibiotics

Compounds	Molecular formula	Active chain	Molecular weight
Amoxicillin (AMOX)	$C_{16}H_{19}N_3O_5S$	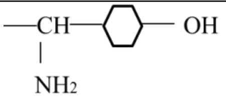	365.4 g/mol
Spiramycin (SPM)	$C_{43}H_{74}N_2O_{14}$	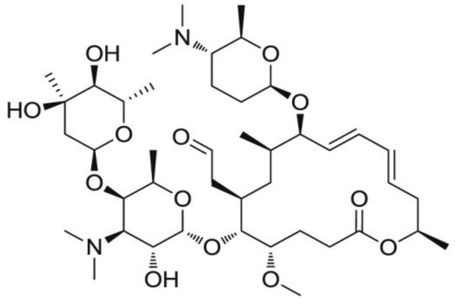	843.1 g/mol

Figure S1. Characterization the antibiotics: (a) FTIR spectrum of amoxicillin, (b) Raman spectrum of amoxicillin, (c) EDX spectrum of amoxicillin; (d) FTIR spectrum of spiramycin, (e) Raman spectrum of spiramycin, (f) EDX spectrum of spiramycin

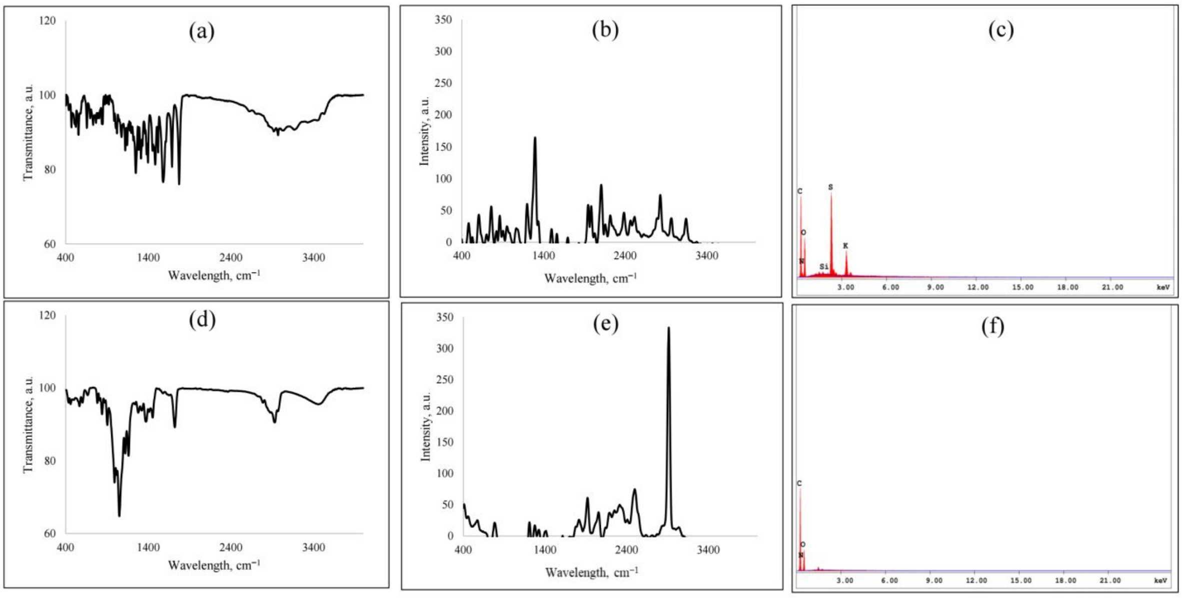

Supplement: Supplementary file 1 — ao2c07927_si_001.pdf [file ao2c07927_si_001.pdf]
